# Supplementary figures and images for: How cortico-basal ganglia-thalamic subnetworks can shift decision policies to increase reward rate
Source: PLoS Comput Biol. 2025 Nov 20;21(11):e1013712. doi: 10.1371/journal.pcbi.1013712 (PMC12668618; doi:10.1371/journal.pcbi.1013712)

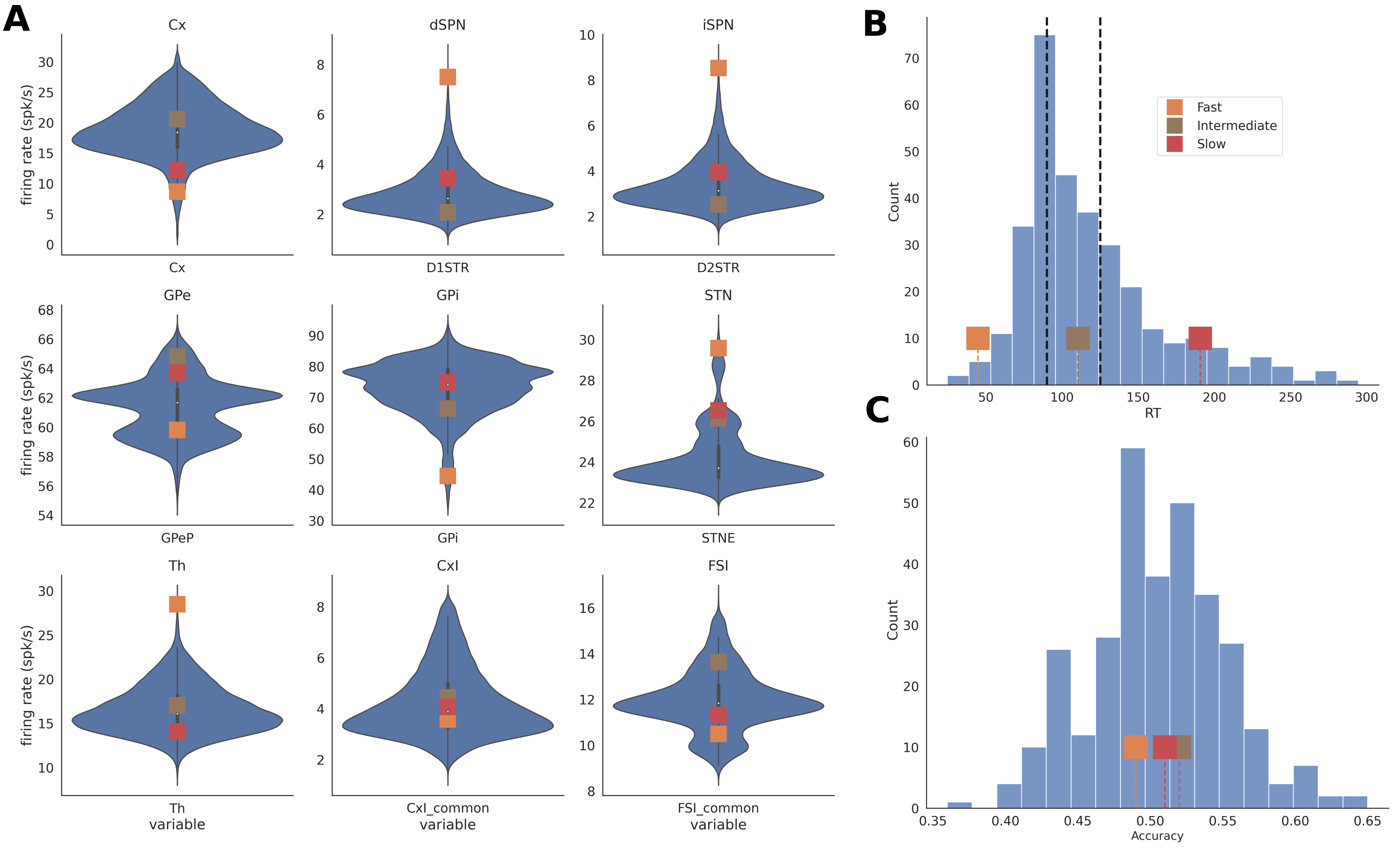

Supplement: S1 Fig — A: The distributions of average firing rates for the 9 CBGT regions based on 300 networks. An average was calculated for each population over the whole simulation time. One example each from three categories of network – fast (orange), intermediate (brown) and slow (red) – are marked on the distribution. B: The networks before plasticity were categorized as fast, intermediate and slow based on a tertile split of the reaction time (RT) distribution (vertical dashed linebs). The RTs for the exemplar fast (orange), intermediate (brown) and slow (red) networks are marked. C: The average accuracies of all 300 networks. The accuracy distribution is centered around 50% (0.5) because the networks had not yet undergone plasticity. (TIFF) [file pcbi.1013712.s006.tif]

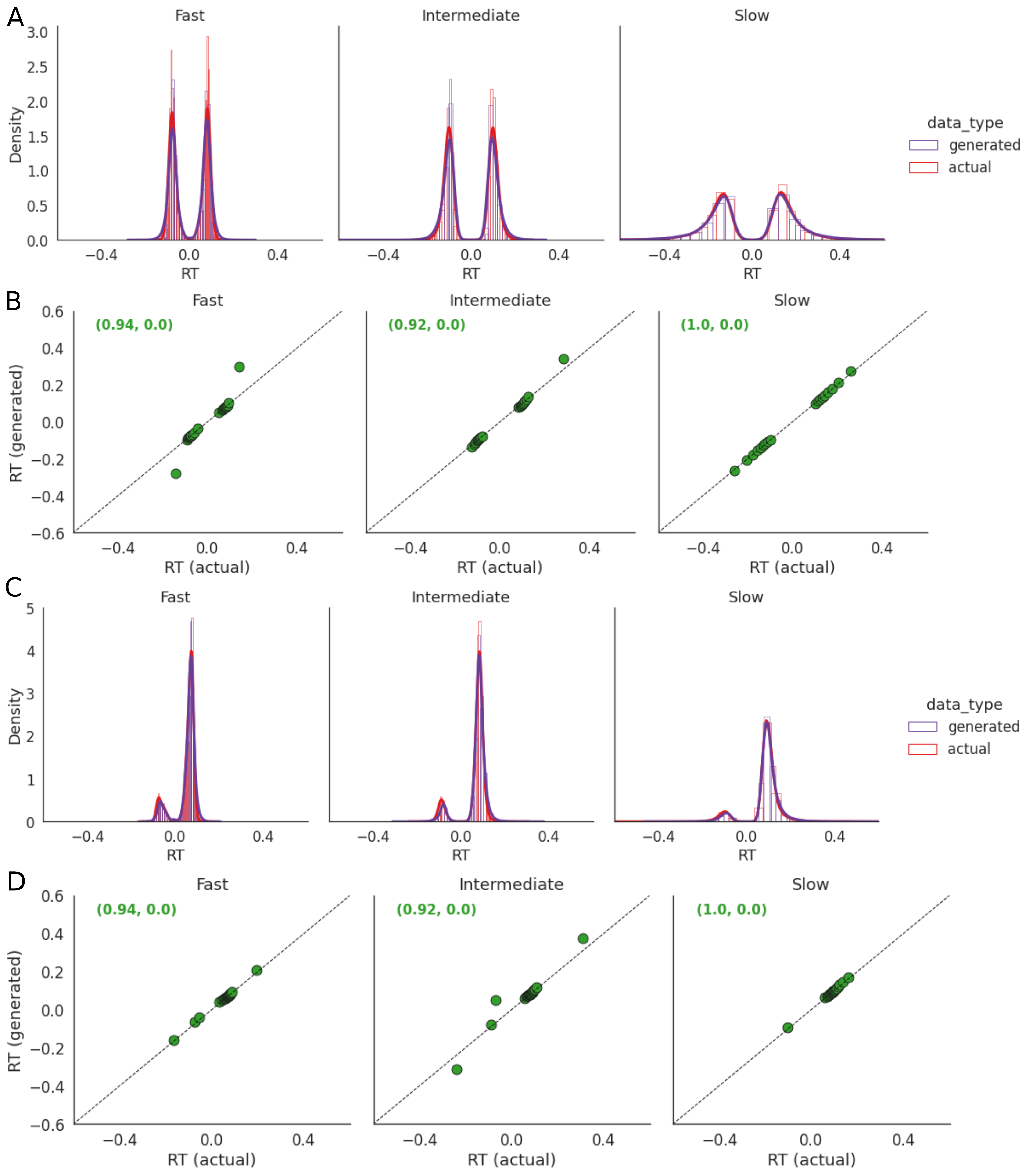

Supplement: S2 Fig — A: The post-predictive choice (i.e., split between positive and negative RTs) and RT distributions from the naive (before plasticity) network simulations (red, “actual”) and distributions generated by the DDM parameters fitted to the data (purple, “generated”) separately for fast, intermediate and slow networks. Note the near-symmetry of the two RT peaks for the two choices (left → positive, right → negative). B: Quantile-Quantile plots for the distributions shown in A for percentiles in steps of 5 (i.e., 5, 10...90, 95). The Pearson correlation and p-value between the actual and generated data are annotated in green. The Pearson correlation was significant for all three network types (0.94, 0.92 and 1.0 for fast, intermediate and slow networks, respectively). C: Same as A but after plasticity with the left choice (positive RTs) rewarded. D: Same as B but after plasticity. (TIFF) [file pcbi.1013712.s007.tif]

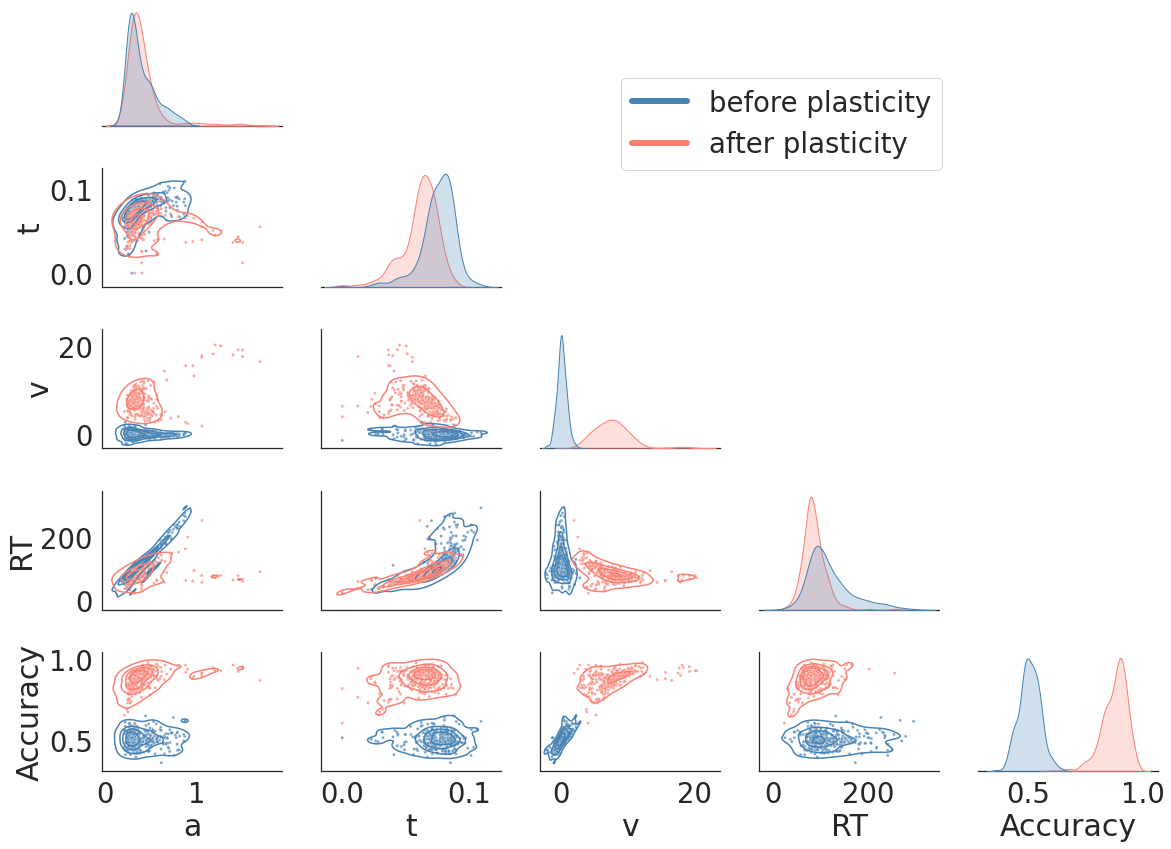

Supplement: S3 Fig — The subplots on the diagonal represent the marginal distributions for DDM parameters (a, t, v) and behavioral features (RT and accuracy). The onset delay (t) shows a decrease, the drift rate (v) shows an increase, RTs show a decrease, and accuracy shows an increase after plasticity. The off-diagonal subplots show the pairwise covariances. (TIFF) [file pcbi.1013712.s008.tif]

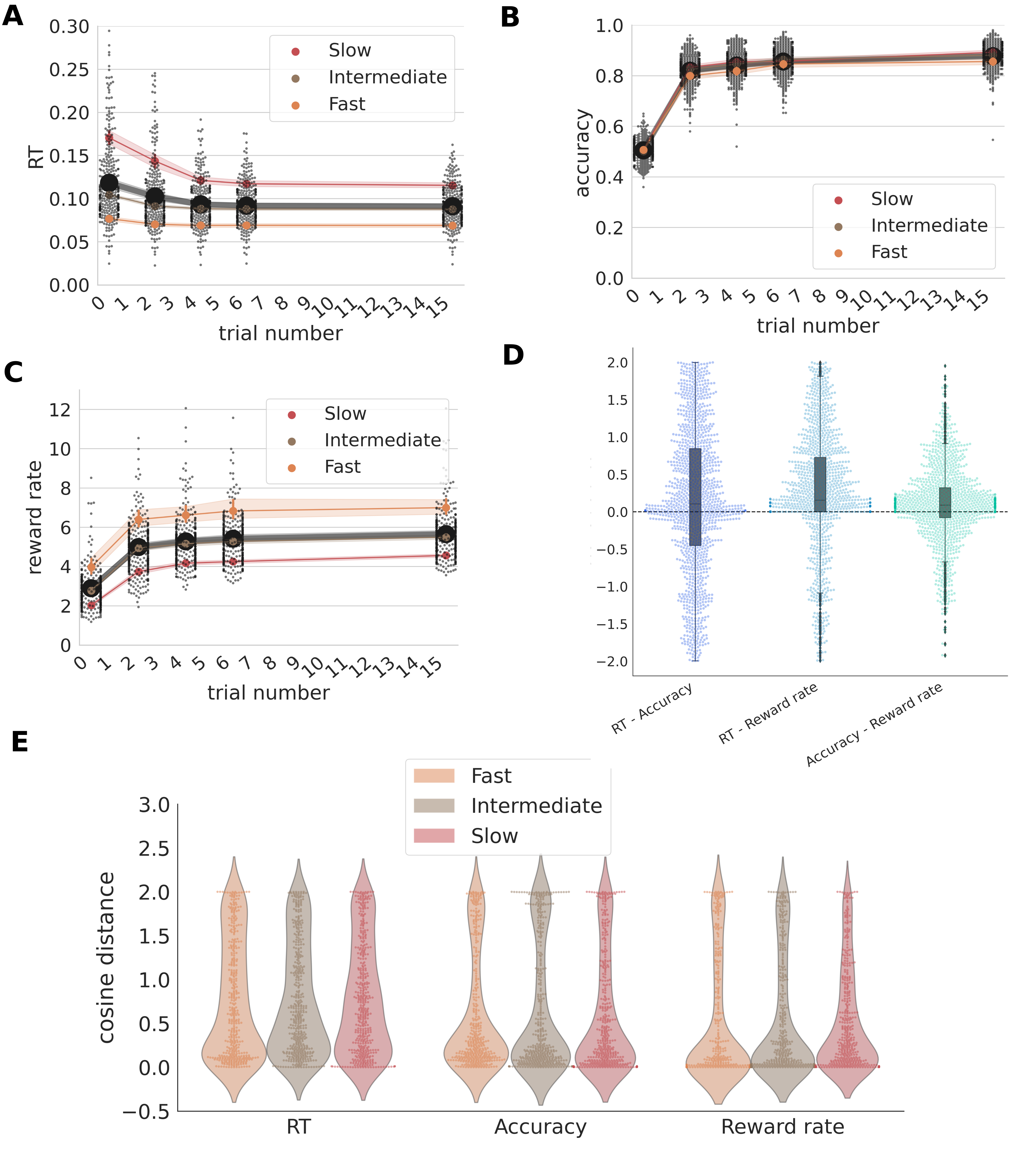

Supplement: S4 Fig — A: Network behavior was assessed after each of 2, 4, 6, 9 and 15 trials. The RTs steadily decreased for all three network categories: fast (orange), intermediate (brown) and slow (red). The average over all 300 networks also showed a steady decrease as shown in black markers and lines. B: The accuracy for the three categories of the networks and the average over all 300 networks increased with plasticity. C: The reward rate for three categories of network and the average over 300 networks increased with plasticity. D: The distribution of differences in cosine distance, measured relative to the direction of greatest increase, for changes in RT vs accuracy, RT vs reward rate, and accuracy vs reward rate for all 300 networks and all stages of plasticity. The comparisons with reward rate yield distributions skewed to significantly above 0, suggesting that the cosine distances are lowest for reward rates. E: Absolute cosine distance distributions shown separately for the three network classes, fast (orange), intermediate (brown) and slow (red). (TIFF) [file pcbi.1013712.s009.tif]

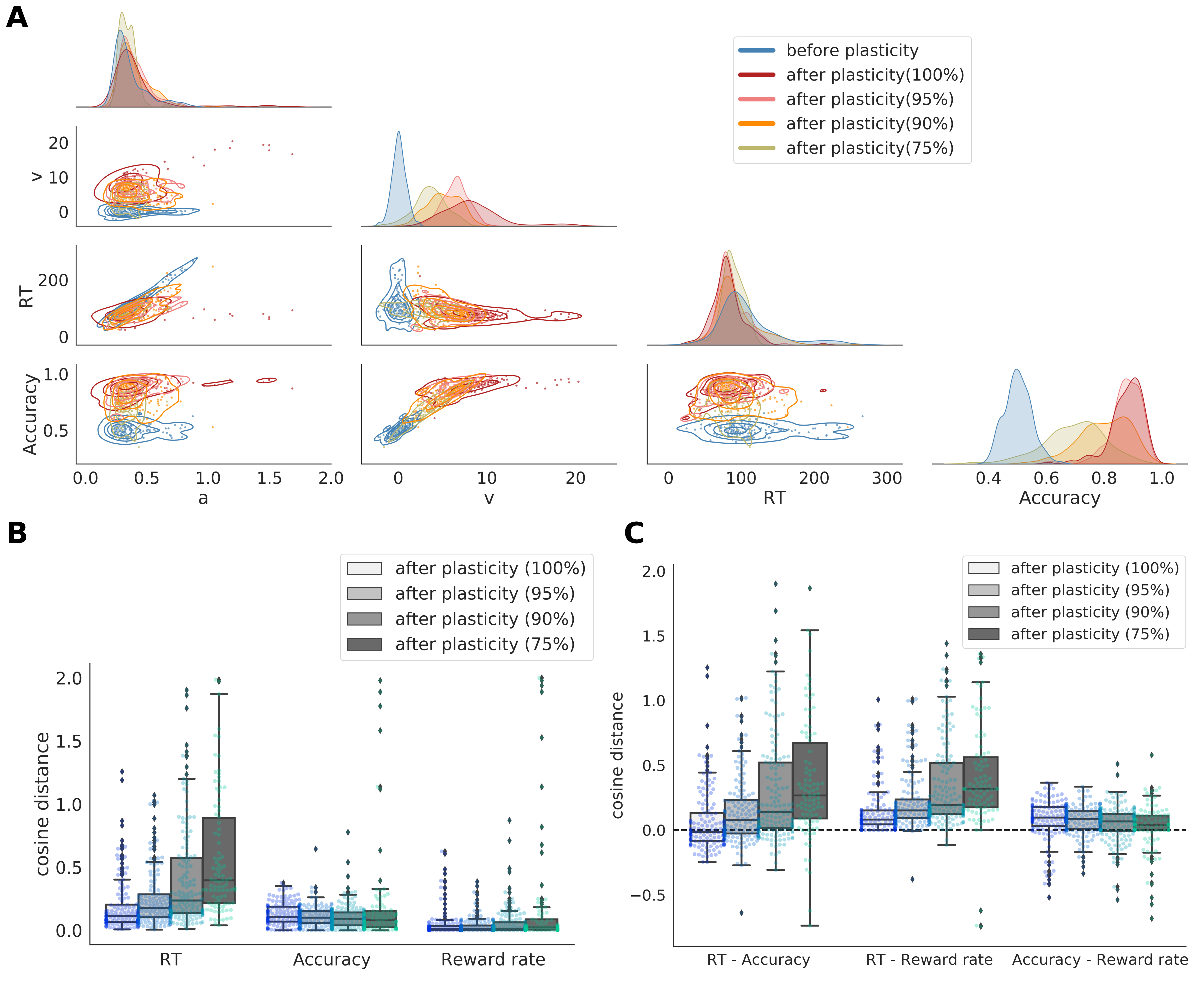

Supplement: S5 Fig — A) DDM (a, v) and behavioral (RT, accuracy) parameters for reward probabilities: 100% (red), 95% (pink), 90% (yellow) and 75% (khaki) measured for 150 networks. The distributions before plasticity are shown in blue. As the conflict increases, accuracy decreases and RTs show a lower average decrease after plasticity B) Cosine distances with respect to the RT (minimization), Accuracy (maximization) and Reward rate (maximization) vectors for the three reward probabilities. The cosine distances scale with the reward probabilities but remain the lowest for reward rates, for 100% reward probabilities and 150 networks in total: F[449,2] = 34.8, p < 0.00001, for 95%: F[449,2] = 89.9, p < 0.00001, for 90%: F[449,2] = 88.8, p < 0.00001 and for 75%: F[449,2] = 25.03, p < 0.00001. C) The distribution of differences in cosine distance, measured relative to the direction of greatest increase, for changes in RT vs accuracy (100%: t(149)= 3.35, p=0.001; 95%: t(149) = 6.72, p < 0.0001; 90%: t(149) = 8.9, p < 0.0001; 75%: t(149) = 7.93, p < 0.0001), RT vs reward rate (100%: t(149)= 9.65, p < 0.0001; 95%: t(149) = 12.42, p < 0.0001; 90%: t(149) = 13.17, p < 0.0001; 75% t(149) = 9.9, p < 0.0001), and accuracy vs reward rate (100%: t(149)= 6.07, p < 0.0001; 95%: t(149) = 8.36, p < 0.0001; 90%: t(149) = 4.06, p < 0.0001; 75%: t(149) = 1.02, p = 0.3) for all the reward probabilities. The comparisons with reward rate yield distributions skewed to significantly above 0 (except for 75%), suggesting that the cosine distances are lowest for reward rates for all the reward probabilities. (TIFF) [file pcbi.1013712.s010.tif]

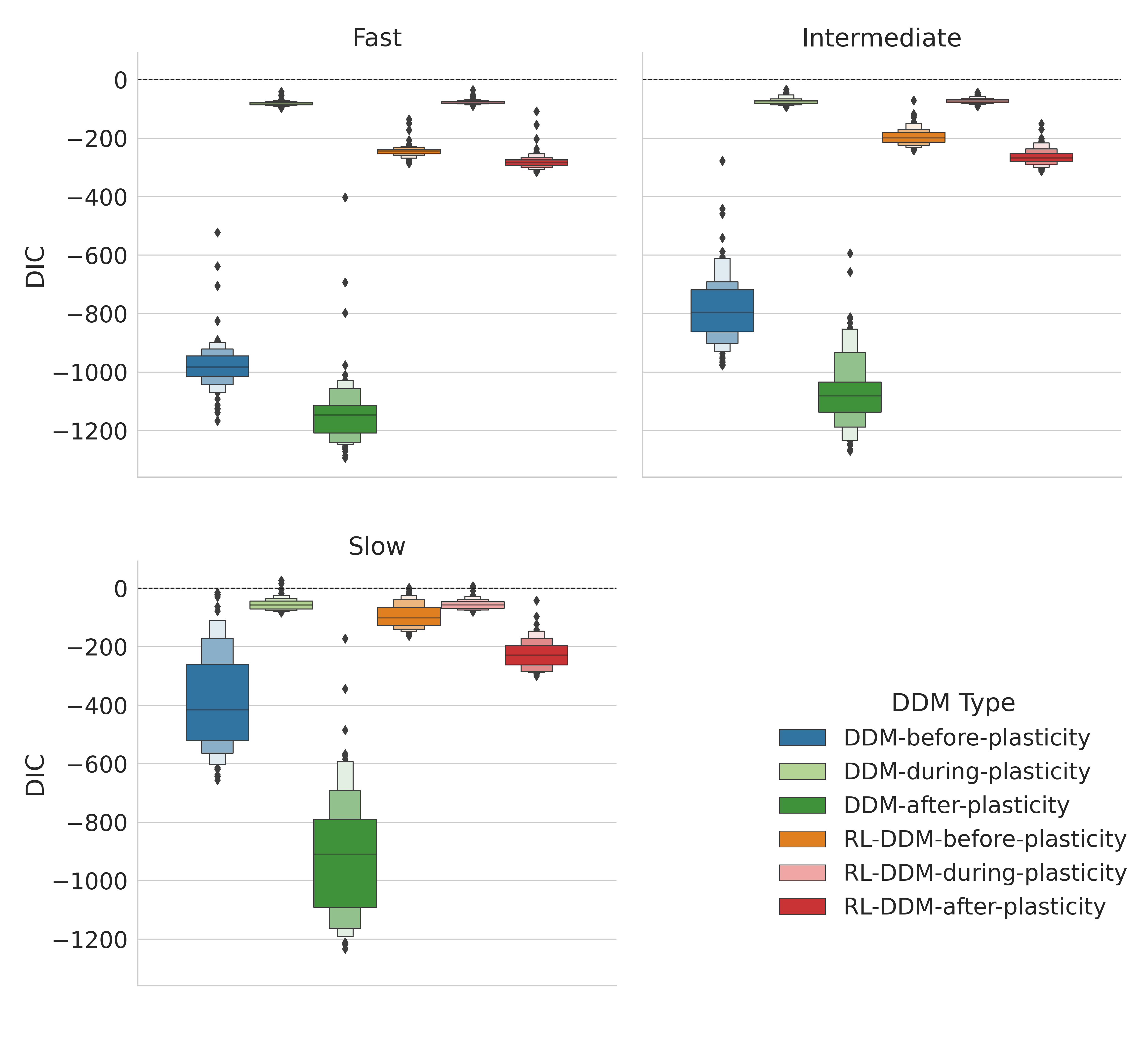

Supplement: S6 Fig — Deviance Information Criterion (DIC) values are compared for DDM and RL-DDM fits across three phases: before, during, and after plasticity. Across all network types (Fast, Intermediate, and Slow) the DDM consistently outperforms the RL-DDM before and after plasticity. However, during plasticity, both models yield comparable fits, indicating that RL-DDM is comparable to DDM in capturing network behavior when plasticity is ongoing, but not once it stabilizes. (TIFF) [file pcbi.1013712.s011.tif]

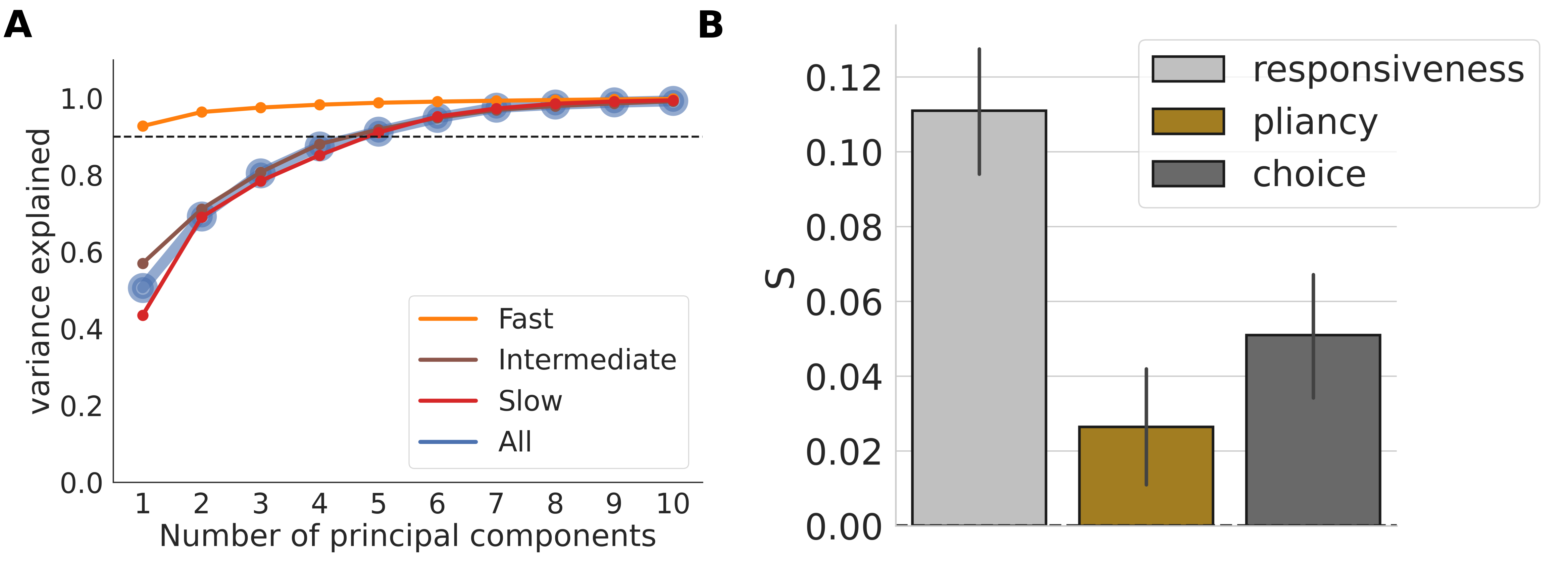

Supplement: S7 Fig — A:) Cumulative variance explained by the first 10 principal components (PC) derived from the changes in firing rates from before to after plasticity. The dashed line indicates 90% of variance explained. The analysis was done for all the networks pooled together (blue line) and separately for fast (orange), intermediate (brown) and slow (red) networks. For all networks pooled together as well as the separated slow and intermediate networks, the first 5 PCs explain more than 90% of the variance, whereas for fast networks 1 PC suffices. B: The weighted sum of the columns of S (see main text, Fig 4B), pooled over all three network classes (fast, intermediate and slow), shows that the observed changes in firing rates correspond to increased loadings of the responsiveness, pliancy and choice ensembles of the CBGT network, to differing extents. (TIFF) [file pcbi.1013712.s012.tif]

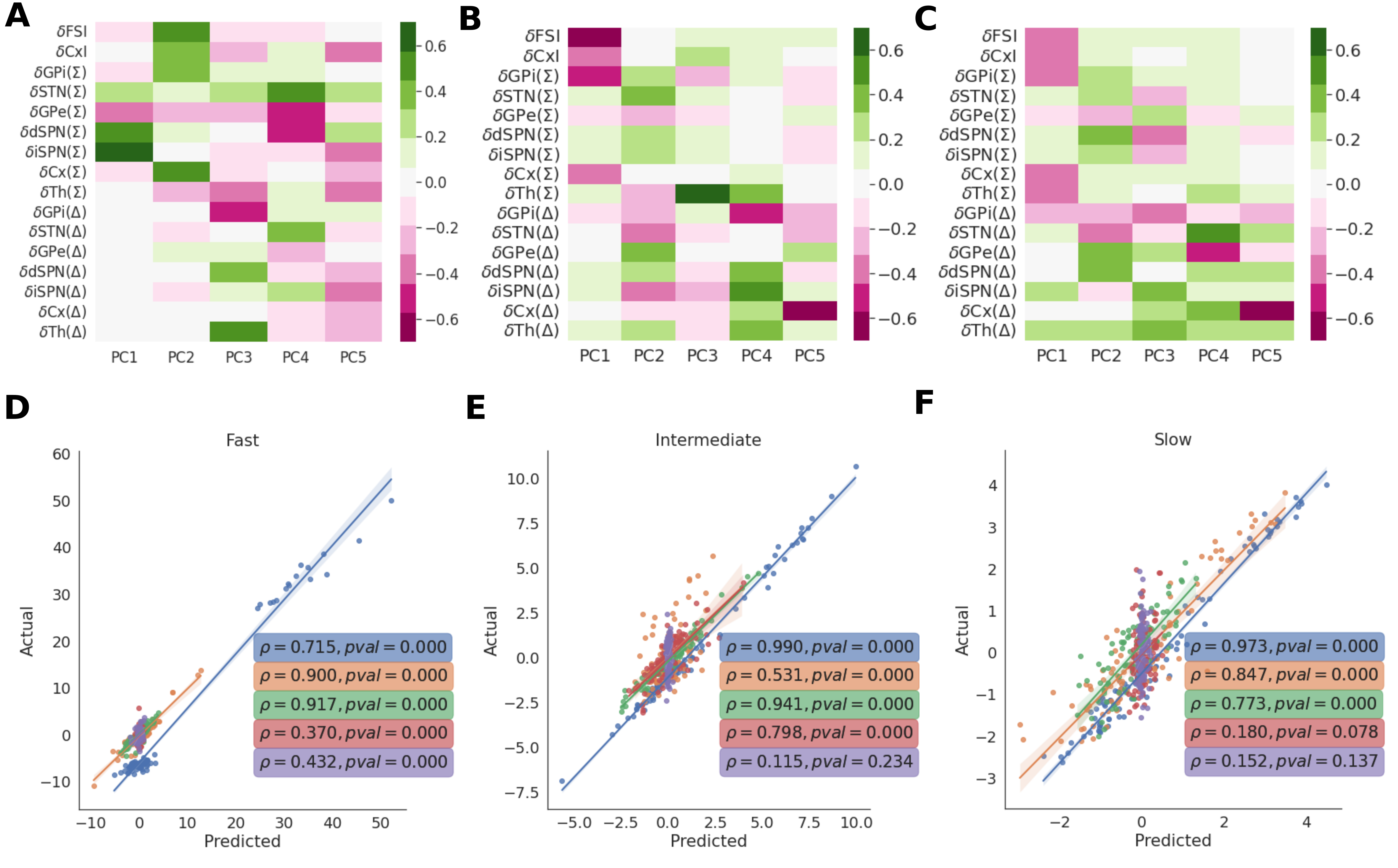

Supplement: S8 Fig — (A) The first 5 PCs for the firing rate changes in the fast networks. Although the 1st PC explains around 90% of the variance for fast networks, we used 5 PCs to calculate S coefficients (Fig 4C) to be consistent with slow and intermediate networks (Supp. S7 FigA). (B,C): Same as (A) for intermediate and slow networks, respectively. (D-F) The dot products of the CCA component vector (C) with each of the 5 columns of S, the least squares solution of P = CS, provide an approximate reconstruction of the 5 PCs of the changes in firing rate from before to after plasticity, (ΔF). The quality of the reconstruction was checked by projecting ΔF onto the original PCs for each network (marked as Actual on y-axis) and comparing the results with the projections of ΔF onto the reconstructed PCs (marked as Predicted on x-axis). The goodness of fit is calculated as the Spearman rank correlation (ρ) between the actual and predicted values. For fast networks (D), the rank correlations (ρ) are high and significant (p < 0.0001) for all of the PCs as shown, suggesting that the reconstruction is excellent. For intermediate networks (E), the rank correlations are significant for all PCs except the 5th PC. For slow networks (E), the rank correlations are significant for all except 4th and 5th PCs. (TIFF) [file pcbi.1013712.s013.tif]

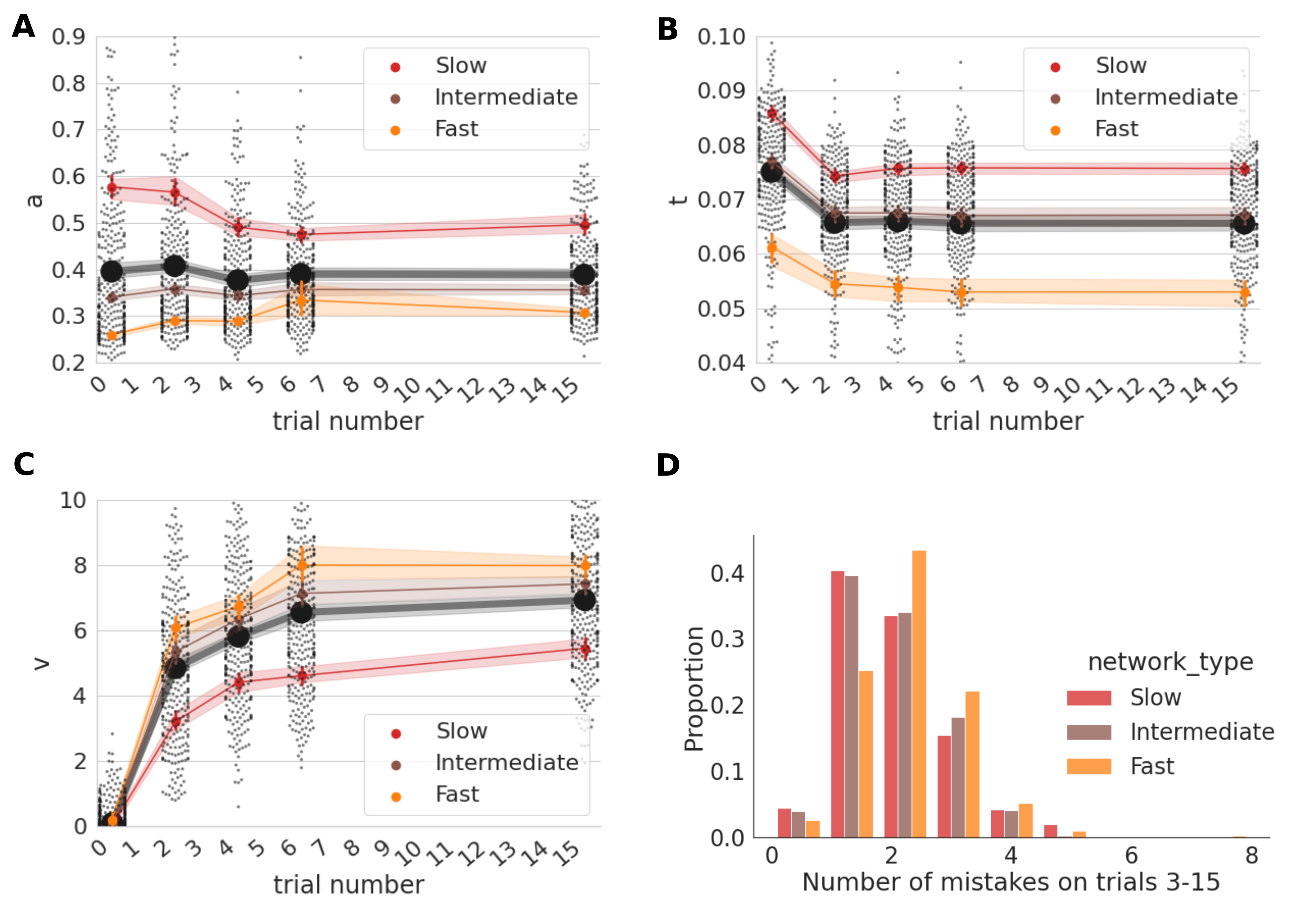

Supplement: S9 Fig — (A) The change in boundary height (a) due to plasticity is dependent on network type: slow networks (red) show a decrease, intermediate (brown) show little change, and fast (orange) networks show a slight increase. The mean over all networks is shown by large black circles. (B) All network types show a decrease in decision onset time (t) due to plasticity. (C) All network types show a strong increase in drift rate (v) due to plasticity. (D) Fast networks make more mistakes on average. The histograms show the proportion of unrewarded (“U”) trials encountered by all the three network classes after the first two plasticity trials. (TIFF) [file pcbi.1013712.s014.tif]

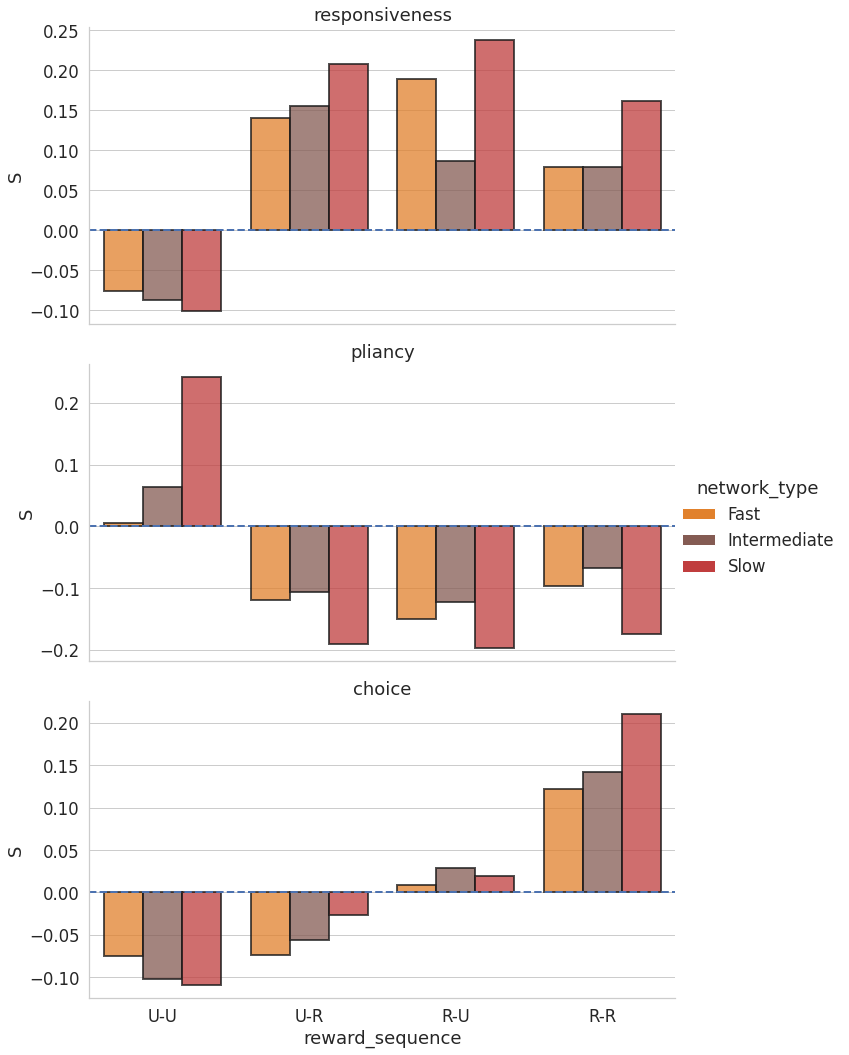

Supplement: S10 Fig — The weighting coefficients S shown in Fig 5A combine the three network types. The separated coefficients here show the same trends as the combined ones. (TIFF) [file pcbi.1013712.s015.tif]

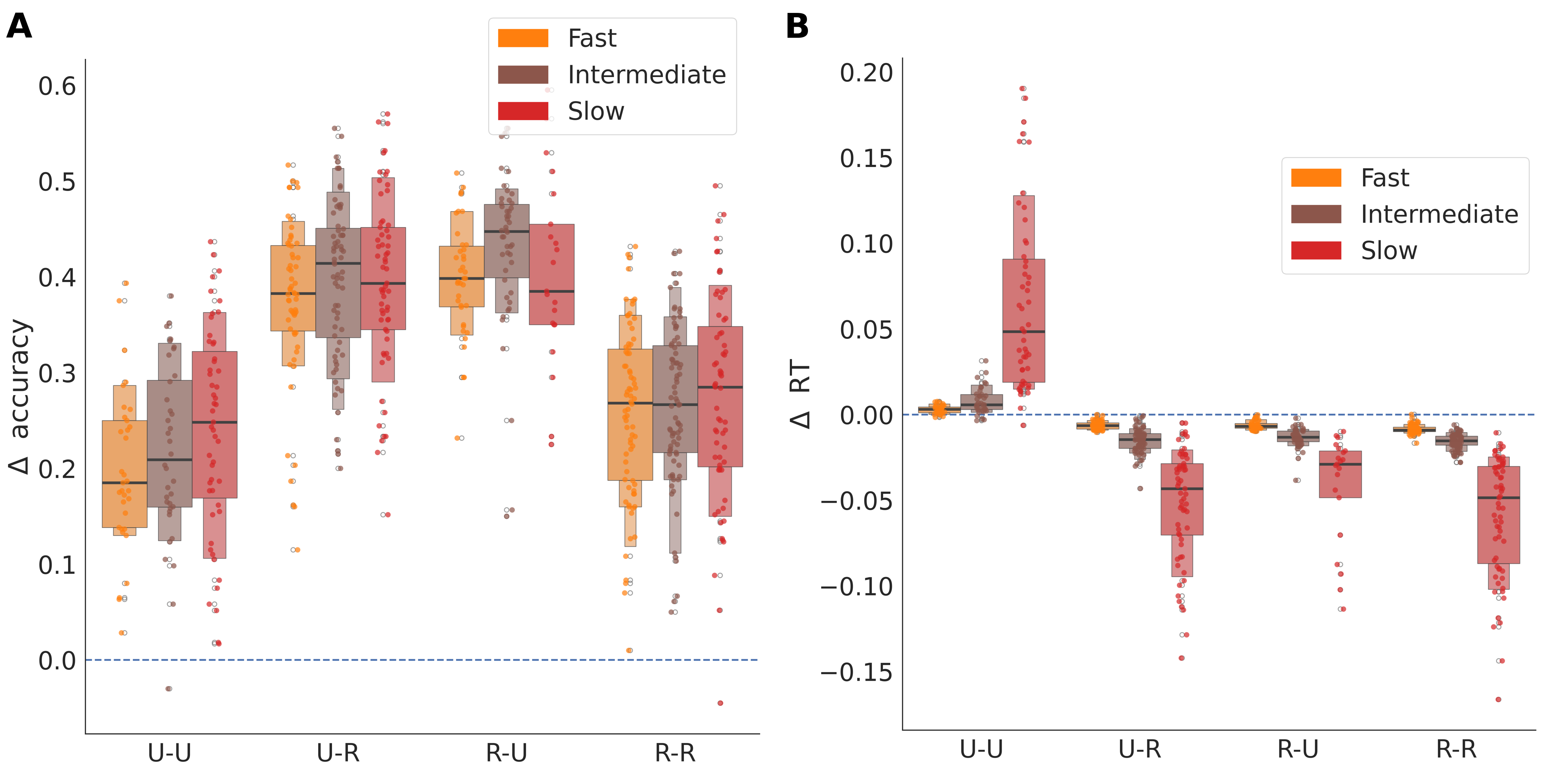

Supplement: S11 Fig — (A) The change in accuracy showed an increase in all cases, but to different extents. The highest increase in accuracy was for one rewarded and one unrewarded trial (U-R and R-U), due to strengthening of the cortico-striatal projection to dSPNs of the optimal choice along with strengthening of cortico-striatal projections to iSPNs of the sub-optimal choice. (B) The change in RTs after plasticity for the four outcome sequences. All sequences involving at least one rewarded trial yielded a decrease in RT, whereas the sequence with two consecutive unrewarded trials (U-U) induced an increase in RT. (TIFF) [file pcbi.1013712.s016.tif]
